# Development of Lentiviral Vectors Pseudotyped With Influenza B Hemagglutinins: Application in Vaccine Immunogenicity, mAb Potency, and Sero-Surveillance Studies

**DOI:** 10.3389/fimmu.2021.661379

**Published:** 2021-05-24

**Authors:** Francesca Ferrara, Joanne Marie M. Del Rosario, Kelly A. S. da Costa, Rebecca Kinsley, Simon Scott, Sasan Fereidouni, Craig Thompson, Paul Kellam, Sarah Gilbert, George Carnell, Nigel Temperton

**Affiliations:** ^1^ Viral Pseudotype Unit, Medway School of Pharmacy, University of Kent & University of Greenwich, Chatham, United Kingdom; ^2^ Department of Physical Sciences & Mathematics, College of Arts and Sciences, University of the Philippines Manila, Manila, Philippines; ^3^ Department of Veterinary Medicine, University of Cambridge, Cambridge, United Kingdom; ^4^ Research Institute of Wildlife Ecology, Veterinary Medicine University, Vienna, Austria; ^5^ Department of Zoology, University of Oxford, Oxford, United Kingdom; ^6^ Faculty of Medicine, Imperial College London, London, United Kingdom; ^7^ The Jenner Institute, University of Oxford, Oxford, United Kingdom

**Keywords:** influenza B, lentiviral (LV) vector, pseudotype viral particles, pseudotype neutralization, serosurveillance

## Abstract

Influenza B viruses (IBV) cause respiratory disease epidemics in humans and are therefore components of seasonal influenza vaccines. Serological methods are employed to evaluate vaccine immunogenicity prior to licensure. However, classical methods to assess influenza vaccine immunogenicity such as the hemagglutination inhibition assay (HI) and the serial radial hemolysis assay (SRH), have been proven to have many limitations. As such, there is a need to develop innovative methods that can improve on these traditional assays and provide advantages such as ease of production and access, safety, reproducibility, and specificity. It has been previously demonstrated that the use of replication-defective viruses, such as lentiviral vectors pseudotyped with influenza A hemagglutinins in microneutralization assays (pMN) is a safe and sensitive alternative to study antibody responses elicited by natural influenza infection or vaccination. Consequently, we have produced Influenza B hemagglutinin-pseudotypes (IBV PV) using plasmid-directed transfection. To activate influenza B hemagglutinin, we have explored the use of proteases in increasing PV titers *via* their co-transfection during pseudotype virus production. When tested for their ability to transduce target cells, the influenza B pseudotypes produced exhibit tropism for different cell lines. The pseudotypes were evaluated as alternatives to live virus in microneutralization assays using reference sera standards, mouse and human sera collected during vaccine immunogenicity studies, surveillance sera from seals, and monoclonal antibodies (mAbs) against IBV. The influenza B pseudotype pMN was found to effectively detect neutralizing and cross-reactive responses in all assays and shows promise as an effective and versatile tool in influenza research.

## Introduction

Seasonal influenza causes severe illness and mortality in high-risk human populations such as children, the elderly, and individuals with underlying morbidity. Influenza viruses belong to the *Orthomyxoviridae* family and are enveloped negative sense single−stranded segmented RNA viruses. Within this family, there are three influenza types that circulate in humans: influenza A, B, and C (1, 2). Influenza type D was recently proposed but it appears to be confined to animal populations (3). Influenza B virus (IBV) causes approximately 25% of all seasonal influenza infections (4) and has also been shown to infect seals (5) and pigs (6). IBV co-circulates with influenza A viruses (IAV) in humans as two distinct lineages, defined as the B/Yamagata/16/88-like viruses and B/Victoria/2/87-like lineages (7, 8). The two B lineages have been shown to cause a comparable proportion of influenza B cases globally, with B/Yamagata more frequent in temperate countries, and B/Victoria in the tropics (9). The two lineages are defined by genetic and antigenic differences of their major surface glycoprotein hemagglutinin (HA). Hemagglutinin (HA) is a homotrimeric type I glycoprotein that is responsible for viral cell attachment and entry into host cells *via* protein-linked sialic acid receptors (10, 11). Sialic acids are a family of carboxylated sugars that constitute the terminal monosaccharides of animal protein glucidic residues (12). If infection was not limited by other factors controlling viral replication, the HA would allow broad viral tropism, since sialic acids are present on almost all cell types regardless of species. These limiting factors are mainly represented by restriction of the influenza polymerase complex activity and activation of influenza HA (10, 13–16). HA is synthesized as a single polypeptide precursor, HA0 (78kDa) that is then cleaved into two subunits HA1 (50−58 kDa) and HA2 (28−22 kDa). HA cleavage, mediated by specific proteases (17), is necessary to expose a fusion peptide in the stalk region, and to permit HA conformational change after viral particle endocytosis. Subsequently, this permits envelope-endosome membrane fusion and the release of the viral core into the cell cytosol (14). However, these factors do not completely limit the ability of influenza viruses to infect different animal species and as is the case with IAV, IBVs cause human infections and have zoonotic and pandemic potential due to their ability to infect non-human mammalian species. Interestingly, these non-human IBVs are antigenically related to human viruses (5) indicating that humans are the natural reservoir of IBV and that they have limited zoonotic potential. In fact, IBV has a lower infection and mortality rate than influenza A and this has resulted in influenza B being relatively understudied (15).

The clinical and public health community has until recently, underestimated the influenza B burden. It is now necessary to raise awareness of influenza B virus infection, by increasing epidemiological surveillance and seasonal vaccination coverage (18). Influenza B strains are included in seasonal vaccines, but there have been instances wherein mismatches between vaccine and circulating strains have left the at-risk population unprotected (19). This may partially be due to IBV branching into two distinct lineages in the 1980’s following a large−scale genomic reassortment event involving all eight influenza B segments (8). These two lineages continue to diverge, and are subject to reassortment and co−circulation, although one tends to dominate over the other for a determinate period of time (7). To resolve the vaccine mismatch issue, quadrivalent vaccines containing one representative strain for each influenza B lineage together with the influenza A H1N1 and H3N2 viruses were developed and are currently licensed (20–22). Despite improved vaccine coverage, epidemiological surveillance and rigorous vaccine testing are still needed (20, 23).

Classical serological assays, such as the hemagglutination inhibition (HI), Single Radial Hemolysis (SRH), and microneutralization (MN) assays are usually employed for influenza surveillance (24). However, several studies have indicated that the HI assay does not quantify Influenza B seroconversion accurately since it underestimates antibody titers (25–27). Although, ether−treatment of the virus during HI was shown to increase sensitivity (28–30), this increase may be insufficient to attain SRH and MN consistency (28, 29, 31). Additionally, classical serological methods do not efficiently detect HA stalk−directed antibodies (32–36), as they are known to be unable to inhibit hemagglutination, and their discovery was hampered by this bottleneck in previous years. HA stalk epitopes are promising targets for influenza ‘universal’ vaccines (37–39). Encouragingly, there is evidence that stalk –directed antibodies are able to neutralize the two influenza B lineages and in some cases, also influenza A viruses (33, 40). This work has increased interest in studying the cross−neutralizing response from an influenza B perspective. Monoclonal antibodies can be employed to delineate these responses, whereupon lineage specific or broader epitopes can be identified and tested, yielding data which would not be possible using polyclonal sera.

In recent years, the use of lentiviral vectors based on the HIV-1 genome pseudotyped with the HAs of influenza A viruses have been shown to be effective for studying antibody responses (32, 41, 42). An advantage of working with these influenza pseudotypes (PV) is the possibility to investigate immune response to influenza without the use of high containment facilities. The use of pseudotypes enables BSL-2 research employing viruses that are not commonly in circulation and have high pandemic potential, as these live viruses are often studied at BSL-3 or higher. Additionally, in vaccine studies, high volumes of serum are required for live virus testing and hemagglutination assays, whereas a very low sample input is needed in assays using pseudotype viruses. These pseudotypes also provide a system to explore the role of HA-head or HA-stalk-directed immune responses. It has been proposed that the lower density of HA expressed on the vector surface permits increased access to HA-stalk epitopes; the proposed major mediator of the heterosubtypic antibody response (32, 43–45).

To address the aforementioned serological assay issues and allow further study of IBV antibody responses, we have developed a panel of lentiviral vectors pseudotyped with IBV HAs. Herein, we describe the optimized production and characterization of this newly established Influenza B PV panel. Additionally, we have investigated the feasibility of using these PV as surrogate antigens in neutralization assays, and to study cross−neutralizing antibody responses.

## Materials and Methods

### Propagation and Maintenance of Cell Lines

Human Embryonic Kidney (HEK) 293T/17 cells were used for PV production, titration and as target cells. They were maintained in Dulbecco’s Modified Eagle Medium (DMEM) (PAN−Biotech P04-04510) supplemented with 15% (v/v) Fetal Bovine Serum (FBS) (PAN−Biotech P30-8500) and 1% (v/v) penicillin−streptomycin (Pen-Strep) (Sigma P4333).

Madin−Darby Canine Kidney (MDCK) and A549 cells were also used as target cells to study IBV PV transduction and tropism. Cells were cultured in DMEM supplemented with 5% FBS and 1% of Pen-Strep and in DMEM/F-12 media (Gibco 21041033) with 10% FBS and 1% Pen-Strep, respectively.

### HA, Protease, and Lentiviral Vector Packaging Plasmid Production

**Figure 1:**
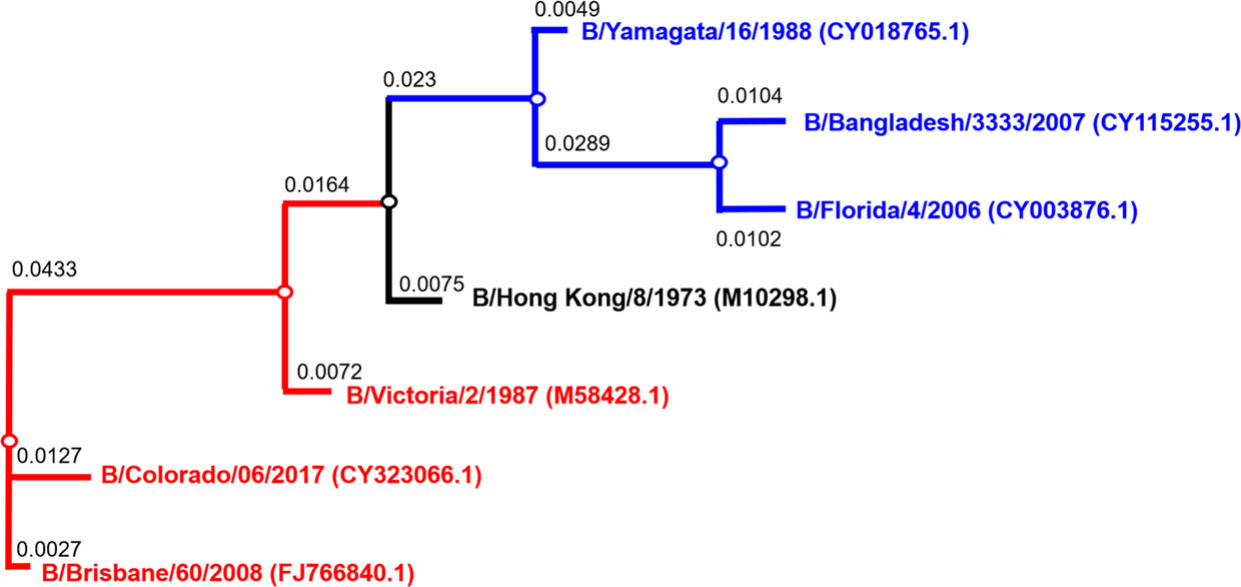
Phylogenetic tree of influenza B HA used for pseudotype production. Phylogenetic tree shows influenza B diverging into two distinct lineages, B/Yamagata (blue) and B/Victoria (red). Accession numbers are reported with the strain name on the tree tips. Nodes are shown at the ends of branches which represent sequences or hypothetical sequences at various points in evolutionary history. Branch lengths indicate the extent of genetic change. The tree generated was constructed with PhyML (48) and graphically elaborated with Archaeopteryx.js (https://sites.google.com/site/cmzmasek/home/software/archaeopteryx-js).

B/Bangladesh/3333/2007 and B/Brisbane/60/2008 HA genes were amplified from cDNA and subcloned into plasmid expression vector pI.18 (46, 47). B/Hong Kong/8/1973, B/Victoria/2/1987, B/Yamagata/16/1988 and B/Florida/4/2006 codon optimized HA genes (**Figure 1**) were synthesized by GenScript and cloned into phCMV1 as previously described (32). B/Colorado/06/2017, B/Phuket/3073/2013, B/Brisbane/60/2008 and B/Washington/2/2019 plasmids were synthesized by GeneArt and cloned into pEVAC. HAT (human airway trypsin-like protease) and TMPRSS2 (type II transmembrane protease serine 2) (49) were cloned in pCAGGS; while TMPRSS4 (type II transmembrane protease serine 4) (50) was cloned in pCMV, and were used to activate influenza B HA during lentiviral vector production. The second-generation packaging construct pCMVΔR8.91 (51), and the self-inactivating lentiviral vectors pCSFLW expressing firefly luciferase and pCSemGW expressing emerald green fluorescence protein (emGFP) were used as reporters for PV production.

### Production of IBV Pseudotypes

For IBV HA in pI.18, pseudotypes were produced in 6-well plates using branched polyethylenimine (PEI) (Sigma-Aldrich 408727) co-transfection of 500 ng pCMVΔR8.91, 750 ng pCSFLW or pCSemGW, and 500 ng of HA-encoding plasmid. Additionally, 125 ng or 250 ng of protease-encoding plasmid was also included in the DNA transfection mix to allow influenza B HA activation and cleavage. For IBV HA in pEVAC, cells were transfected using Opti-MEM™ (Thermo Fisher Scientific 31985062) and FuGENE^®^ HD Transfection Reagent (ProMega E2312*)* with 10 ng HA encoding plasmid, 375 ng pCSFLW, 250 ng pCMVΔR8.91, and 2.5-10 ng of HAT or T4 proteases. IBV PV were also produced in the absence of protease-encoding plasmid (Δ protease), and these served as controls. At least 8 hours post-transfection, 1 unit of exogenous Neuraminidase (Sigma N2876) was added. PV were collected 48 hours after transfection of HEK293T/17, passed through a 0.45 µm membrane filter, and stored at -80°C.

### Titration of IBV PV and Transduction of Different Cell Lines

Titration experiments of firefly luciferase-expressing or emerald GFP PV were performed in Nunc™ F96 MicroWell™ white or transparent polystyrene plates (Thermo Fisher Scientific), respectively. The PV production titer was evaluated by transduction of HEK293T/17, MDCK, and A549 cells, with 2−fold serial dilutions of PV in a total volume of 100 µL. As a control for HA cleavage, Δ protease influenza B PV were also activated by addition of 1 mg/mL (TPCK)-trypsin (Sigma T1426) to a final concentration of 100 µg/mL per well. These wells were incubated at 37°C, 5% CO_2_ for 30 minutes followed by addition of 50 µL trypsin neutralizing solution (Lonza CC-5002) to each well. For all samples and controls, 1.5x10^4^ HEK293T/17 cells were added to each well of the 96-well plate. Plates were incubated for 48 hours at 37°C, 5% CO_2_. Afterwards, firefly luciferase gene expression was evaluated and quantified using the Bright−Glo™ assay system (Promega E2620) and GloMax Multi detection system luminometer (Promega) as shown previously (52). Influenza B PV produced with emerald GFP were also tested, using an equivalent protocol, for their ability to transduce HEK293T/17, MDCK, and A549 cells.

### Serum Samples and Monoclonal Antibodies

Antisera against B/Brisbane/60/2008 (NIBSC 11/136) was obtained from the National Institute for Biological Standards and Control (NIBSC) and used as the primary antibody in western blot experiments and positive control in neutralization assays. Post-vaccination mouse sera were obtained from the University of Cambridge as part of an ongoing influenza vaccination study and were employed to determine responses of different antigens used for vaccination against IBV PV. Human sera obtained during the clinical trial NCT00942071 (53) were used in PV neutralization assays (pMN). These sera were collected from adults aged 50 years and over pre− and post− administration of an influenza split virion vaccine (Sanofi Pasteur MSD, France) that contained HAs of A/California/7/2009 (H1N1), A/Perth/16/2009 (H3N2), and B/Brisbane/60/2008. Nine subjects received an injection of Modified Vaccinia Ankara (MVA) vectors expressing the A/Panama/2007/1999 (H3N2) nucleoprotein (NP) and matrix protein 1 (M1) (MVA−NP+M1) antigens as a single fusion protein (53, 54). The remaining subjects (n=8) received a trivalent inactivated vaccine (TIV) and saline placebo. These sera were previously analyzed (53) in an HI assay against B/Brisbane/60/2008. Seal serum samples (n=8) were obtained from the University of Veterinary Medicine, Vienna, Austria, as part of a wildlife and conservation study of seals caught in the Caspian Sea (CS) from 2006-2010 at the Europe/Asia border. We obtained previously characterized neutralizing monoclonal antibodies (55) from the Division of Viral Products, Food and Drug Administration, USA. Monoclonal antibodies designated 5A1 specific to the B/Victoria lineage, 3E8, specific to the B/Yamagata lineage, and 2F11 specific to both B/Victoria and B/Yamagata lineages are used herein to evaluate PV neutralization activity (55).

### Western Blotting

Briefly, 2 ml of PV was centrifuged at 3000*xg* at 4°C for 24 hours. Supernatant was removed and resuspended in 150 µL OptiMEM™. SDS−PAGE of reduced and denatured influenza B PV was carried out in 4−15% Mini−PROTEAN^®^ TGX™ precast polyacrylamide gels (Bio−Rad 4561024) following manufacturer’s instructions. The gel was transferred onto an Immuno−Blot^®^ low fluorescence polyvinylidene fluoride membrane (PVDF) (Bio−Rad 1620260) for 60 minutes at 100 V. The membrane was blocked overnight with blocking buffer (10% (w/v) dried milk, 0.01% (v/v) Tween20−PBS). The anti−B/Brisbane/60/2008 serum (NIBSC 11/136) was used as the primary antibody, diluted 1:500 in blocking buffer. The membrane was washed with 0.01% (v/v) Tween20−PBS prior to addition of secondary antibody, IgG Dylight^®^800 antibody diluted 1:20000 in blocking buffer. After washing to remove unbound secondary antibody, the PVDF membrane was visualized using the Odyssey^®^ Sa Infrared Imaging System (LI−COR Bioscience) at 800 nm.

### Pseudotype Microneutralization Assay (pMN)

The neutralization activity of anti−B/Brisbane/60/2008 serum (NIBSC 11/136) (starting dilution 1:1000), post-vaccination mouse sera (University of Cambridge), sera from the NCT00942071 (53) human clinical trial, and mAbs from Food and Drug Administration (USA) were evaluated against different influenza B pseudotypes produced in this study by performing the pMN according to established protocol (52). Briefly, 2−fold serial dilutions of serum or mAbs were performed in a Nunc™ F96 MicroWell™ white polystyrene plate. The plate was centrifuged for 1 min at 500*xg* and 50 µL of influenza B PV solution with a concentration 1×10^6^ relative luminescence units (RLU) was added to each well. The plate was again centrifuged for 1 min at 500*xg* and incubated at 37°C, 5% CO_2_. Afterwards 50 µL of 1.5×10^4^ HEK293T/17 cells were added to each well and after a final centrifugation for 1 minute at 500*xg*, the plate was incubated at 37°C, 5% CO_2_ for 48 hours. Firefly luciferase gene expression was evaluated as previously described. Additionally, the neutralization activity of a panel of seal sera was also evaluated using a starting dilution of 1:100 (**Supplementary Table 1**). Human positive controls were obtained from Prof. Emanuele Montomoli at the University of Siena (starting dilution 1:1000). The negative control used was FBS (starting dilution 1:100).

### Statistical Analysis

All analyses were performed with GraphPad Prism 8.12 for Windows (GraphPad Software). A non−parametric Analysis of variance (ANOVA) was carried out to determine whether differences exist among the means of three or more groups when compared to control. If differences were found, the post-hoc Tukey multiple comparison test was performed to identify if there is a significant difference between pairs of experimental groups. Wilcoxon matched−pairs signed rank test was used to assess statistical significance between day 0 and day 21 in the clinical trial as the data did not follow Gaussian distributions. Clinical trial data were also then stratified based on vaccine regimen: TIV + placebo or TIV + MVA−NP+M1, to evaluate the effect of MVA−NP+M1 in broadening the heterosubtypic antibody response. To avoid multiple comparisons, statistical analysis was performed using the IC_50_ fold−increase between TIV + placebo and the TIV + MVA−NP+M1 groups in a Mann−Whitney U test.

### Bioinformatic Analysis

Influenza B HA gene sequences that were used in PV production were used to calculate percentage identity between amino acid sequences, and for phylogenetic analysis. First, HA−encoding sequences were downloaded from the Influenza Virus Resource database (IVRD). Then, percentage identity between amino acid sequences was calculated by pair−wise alignment using Jalview (56).

For phylogenetic analysis, codon−based alignment was performed using the MUSCLE algorithm (57) in Molecular Evolutionary Genetics Analysis (MEGA) (58). The phylogenetic “Quick Tree” model used PhyML (48) and pre-defined settings by the Influenza Resource Database of the National Institute of Allergy and Infectious Diseases (NIH/DHHS) to infer phylogenies based on sequences (https://www.fludb.org/brc/tree.spg). The tree generated was then graphically elaborated with Archaeopteryx.js (https://sites.google.com/site/cmzmasek/home/software/archaeopteryx-js) in phyloXML and format.

## Results

### Co-Transfection With a Protease-Encoding Plasmid Produces High Titer Influenza B PV

**Figure 2:**
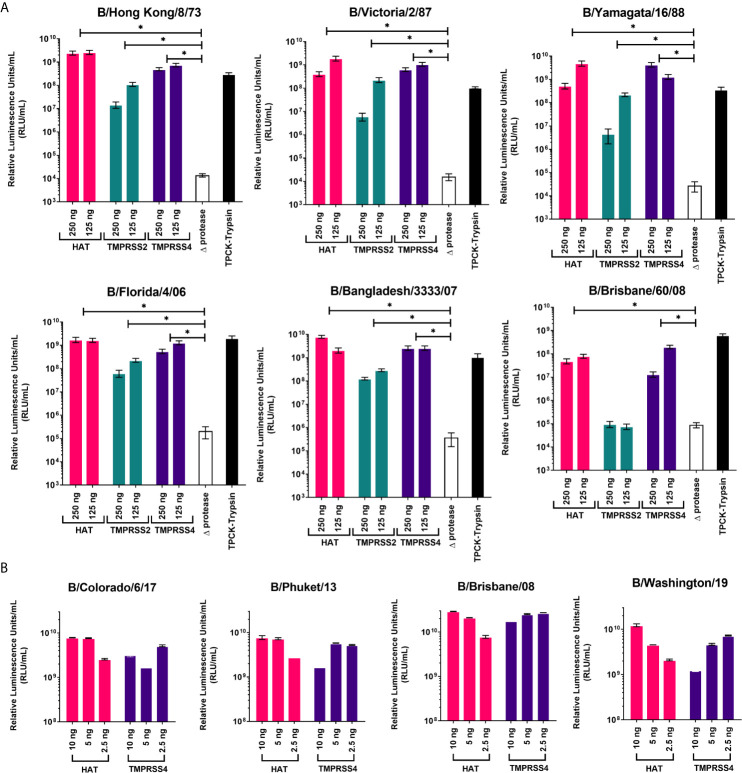
Production of influenza B pseudotypes employing different proteases. Various quantities of proteases were co-transfected with gag-pol packaging construct, firefly luciferase and influenza B HA-encoding plasmids in pI.18 **(A)** or pEVAC **(B)**. **(A)** For transfection employing pI.18 HA plasmids, HAT, TMPRSS2 and TMPRSS4 were used in 250 and 125 ng quantities. Pseudotypes produced in the absence of protease (Δprotease) and those activated post-transfection and pre-titration with TPCK-Trypsin are also included. Plates were read using the Bright-Glo™ assay system (Promega) and GloMax Multi detection system luminometer (Promega). **(B)** For transfection employing pEVAC plasmids, only HAT and TMPRSS4 proteases in the range of 2.5 ng-10 ng were used. Titration plates were then read using the GloMax^®^ Navigator (ProMega) using the Promega GloMax^®^ Luminescence Quick-Read protocol. For both **(A, B)**, pseudotype virus titers are expressed in relative luminescence units/mL (RLU/mL). Significant differences between titers obtained for each PV grouped into protease used and Δprotease are shown *via* brackets (*p=<0.05). Error bars show mean and standard deviation of four replicates.

Proteases were previously reported to be necessary for production of high titer influenza A pseudotypes (50, 59). Here we have employed three different proteases that are associated with influenza A HA cleavage: HAT, TMPRSS2, and TMPRSS4, to show how they will affect IBV PV production. We have used fixed amounts of HA-encoding plasmid, packaging construct, and lentiviral reporter plasmids, only varying the type and amount of protease-encoding plasmid in the transfection. For IBV HA in pI.18, the highest titers of around 10^9^ RLU/mL were achieved using HAT as protease, except for B/Brisbane/60/08 which achieved its highest titer of ~10^8^ RLU/mL with TMPRSS4 (**Figure 2A**). For all PV, co-transfection with TMPRSS2 achieved the lowest titers, ~10^5^-10^8^ RLU/mL, a log down from titers produced *via* HAT (**Figure 2A**). In the absence of protease (Δprotease), influenza PV are still produced, but titers only reached ~10^5^ RLU/mL at best, at least 3 logs lower than PV produced in the presence of protease. These PV (Δprotease) were added with TPCK-Trypsin post-production and prior to titration to activate HA cleavage. PV titers increased at least 3-logs compared to Δprotease titers (**Figure 2A**). We co-transfected with HAT and TMPRSS4 proteases to produce PV from IBV HA in pEVAC. For transfections with the IBV HA pEVAC plasmids, we used significantly less plasmid DNA, 10 ng of the IBV HA plasmid, 250 ng of the packaging plasmid, 375 ng of the luciferase reporter plasmid, and 2.5-10 ng of protease-encoding plasmid. We obtained titers of ~10^10^ RLU/mL for all PV produced *via* co-transfection with HAT, and 10^9^-10^10^ RLU/mL with TMPRSS4 co-transfection (**Figure 2B**). The difference in titers observed for B/Brisbane/60/2008 produced may be attributed to the employment of a different plasmid backbone (pI.18 vs pEVAC), and the transfection agents used. Results indicate that transduction titers of different influenza B PV are dependent on the type of protease used and on the quantity of protease-encoding plasmid employed in transfection, with amounts being adjusted to produce optimal titers.

### Proteases Activate Influenza B PV *via* HA Cleavage

The HA of both IAV and IBV are synthesized as an inactive precursor HA0, and its proteolytic cleavage into the mature HA1/HA2 form is a prerequisite for the low pH-induced, irreversible HA conformational change that is essential for virus infectivity (10, 14, 49, 60, 61). To confirm that the addition of an extracellular protease during transfection mediates HA cleavage, a western blot was performed on the B/Brisbane/60/2008 (from pI.18) PV produced. In all B/Brisbane/60/2008 PV produced with the addition of HAT, TMPRSS2 and TMPRSS4, bands corresponding to HA1 (~55kDa) can be observed (**Supplementary Figure 1**). Darker and heavier HA1 bands are observed for PV produced with HAT and TMPRSS4 compared to TMPRSS2. These band intensities correlate with PV titers (**Figure 2A**). Interestingly, PV HA activated by HAT, TMPRSS2 and TMPRSS4 present heterogeneous glycosylation characteristics: more than one band between 50 kDa and 60 kDa, all corresponding to HA1, are present, whereas in the Δprotease PV before and after TPCK−trypsin treatment, only a single band at ~55 kDa is observed (**Supplementary Figure 1**). This band at ~55 kDa shows limited HA0 cleavage and probable HA activation even without proteases (**Supplementary Figure 1**). It is also observed that the HA cleavage mediated by proteases can be reproduced through TPCK−trypsin treatment of the Δ protease PV, as the HA0 band was replaced by bands corresponding to the HA cleavage products (**Supplementary Figure 1**).

### Influenza B PV Can Transduce Different Cell Lines

**Figure 3:**
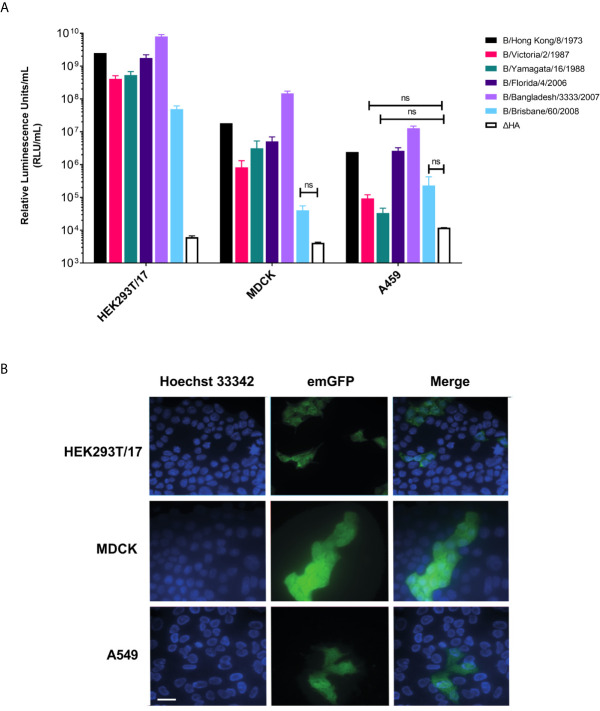
Influenza B pseudotypes are expressed in different cell lines. **(A)** Titers of influenza B pseudotypes expressing firefly luciferase in HEK293T/17, MDCK, and A549 cells are shown in RLU/mL as the mean and standard deviation of 4 replicates. A ΔHA control representing non-HA pseudotyped virus was included for comparisons. No significant difference between PV titers and ΔHA is indicated by brackets (*n.s. p>0.05*). **(B)** Influenza B lentiviral pseudotype B/Florida/4/2006 containing an emerald GFP reporter is shown to successfully transduce HEK293T/17, MDCK and A549 cells. Scale bar = 6 µm.

We tested whether IBV pseudotypes can transduce HEK293T/17, MDCK, and A549 cells at high titer. Employing transfection with pCSFLW (firefly luciferase) (**Figure 3A**) and emGFP (**Figure 3B**), it was found that all IBV pseudotypes are able to transduce all cell lines. It was shown that the IBV PV transduces HEK293T/17 cells more effectively than the classical influenza producing cell lines, MDCK and A549. Titers in HEK293T/17 cells are in the range of 5x10^7^-10^10^ RLU/mL (**Figure 3A**) whilst titers were in the range of 10^4^-10^8^ RLU/mL for pseudotypes using both MDCK and A549 as target cells (**Figure 3A**). Subsequently, we utilized HEK293T/17 cells for production, titration and neutralization assays involving IBV PV in this study.

### Influenza B Pseudotypes as Tools to Assess Vaccine Immunogenicity and for Serological Surveillance

**Figure 4:**
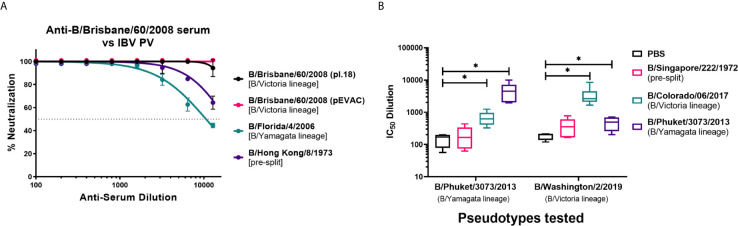
Neutralization of influenza B pseudotypes by reference antisera and mouse sera from influenza vaccination studies. **(A)** Neutralization of pseudotypes from pI.18, B/Brisbane/60/2008, B/Florida/4/2006, and B/Hong Kong/8/1973 against reference antisera anti-B/Brisbane/60/2008 (NIBSC 11/36) was measured by a luciferase reporter assay. Reference sera was serially diluted two-fold from a starting dilution of 1:100. 1.0x10^6^ RLU of IBV PV was then added to each well. For all plots, each point represents the mean and standard error of four replicates per dilution. **(B)** *In vitro* neutralization of influenza B pseudotypes from pEVAC, B/Phuket/3073/2013 and B/Washington/2/2019 by mouse sera vaccinated with Influenza B HA from B/Singapore/222/1972, B/Colorado/6/2017, B/Phuket/3073/2013, and PBS (negative control). Neutralization was determined *via* a luciferase-reporter assay and given as IC_50_ dilution values (In this case, IC_50_ is half maximal inhibitory serum dilution). Significant difference between the means of the IC_50_ values of the groups vaccinated with IBV HA against the PBS negative control group are shown *via* brackets (*p<0.05). Interleaved box plots show minimum, maximum, and mean values. Error bars show standard deviation of replicates. For mice vaccinated with B/Singapore/222/1972 HA, n = 5 mice/group; n = 6 mice/group for mice vaccinated with B/Colorado/6/2017 HA, B/Phuket/3073/2013 HA, and PBS.

We have investigated the utility of influenza B pseudotypes as substitutes for live viruses in influenza neutralization serological studies. We employed standard reference antisera, mouse sera from vaccination studies, human sera from an influenza clinical trial, and sera from seals, a known natural host of influenza B, to test the IBV PV we produced in this study. First, we tested if three of the six IBV PV from pI.18 we have generated (**Figure 2A**) and one IBV PV from pEVAC (**Figure 2B**) could be neutralized by anti-B/Brisbane/60/2008 HA serum (NIBSC 11/36). We chose to test B/Brisbane/60/2008 from the B/Victoria lineage, B/Florida/4/2006 from the B/Yamagata lineage, representing strains of co-circulating influenza B lineages previously or currently used in influenza seasonal vaccination, and B/Hong Kong/8/1973, as a representative strain circulating before the influenza B lineage division (**Figure 1**). It was found that the reference serum employed completely neutralized all the IBV PV tested up to the dilution 1:1600 (**Figure 4A**). The anti-serum neutralized the homologous B/Brisbane/60/2008 most efficiently, followed by B/Hong Kong/8/1973, which shares 94.9% amino acid identity with B/Brisbane/60/2008, and B/Florida/4/2006 (93.5% amino acid identity) (**Figures 1**, **4A**). There was no difference in neutralization of B/Brisbane/60/2008 PV produced from either pI.18 or pEVAC when used in the same input RLU by anti-B/Brisbane/60/2008 HA serum (NIBSC 11/36) (**Figure 4A**). As such, the PV neutralization assay (pMN) with reference antisera was able to differentiate between the different PV, showing correlation between amino acid identity and neutralization efficacy.

Pre-clinical utility: We have obtained sera from Influenza B vaccination studies in mice at the University of Cambridge, and we have tested the ability of these mouse sera to neutralize the IBV pseudotypes, B/Phuket/3073/2013 from the B/Yamagata lineage and B/Washington/2/2019 from the B/Victoria lineage, that we produced using pEVAC-HA (**Figure 2B**). Naïve mice were vaccinated with the HA of B/Singapore/222/1972 (pre-split IBV), B/Colorado/06/2017 (B/Victoria lineage), B/Phuket/3073/2013 (B/Yamagata lineage), and PBS (negative control) to elicit an immune response against influenza B. Mouse terminal bleeds were taken, and sera were run on pMN assays to obtain IC_50_ data and assess vaccine immunogenicity. Post-vaccination sera from groups given B/Colorado/06/2017 and B/Phuket/3073/2013 were able to neutralize both IBV pseudotypes tested regardless of lineage (**Figure 4B** **)** compared to the PBS control (*p<0.05). Vaccination with B/Colorado/06/2017 (B/Victoria) showed highest neutralization against the B/Victoria lineage PV, B/Washington/2/2019. Similarly, vaccination with B/Phuket/3073/2013 (B/Yamagata) neutralized the B/Phuket/3073/2013 PV better than the B/Victoria lineage PV (**Figure 4B**). Mice vaccinated with pre-split B/Singapore/222/1972 was unable to neutralize both IBV with IC_50_ dilutions showing no significant difference (*p>0.05*) to the mice vaccinated with PBS (**Figure 4B**). IC_50_ dilutions were in the range of 10^2^ – 10^5^ for mice vaccinated with either B/Colorado/06/2017 or B/Phuket/3073/2013 against both IBV PV (**Figure 4B** **)**. indicating development of an immune response post-vaccination that can be effectively assessed *via* the PV neutralization assay.

**Figure 5:**
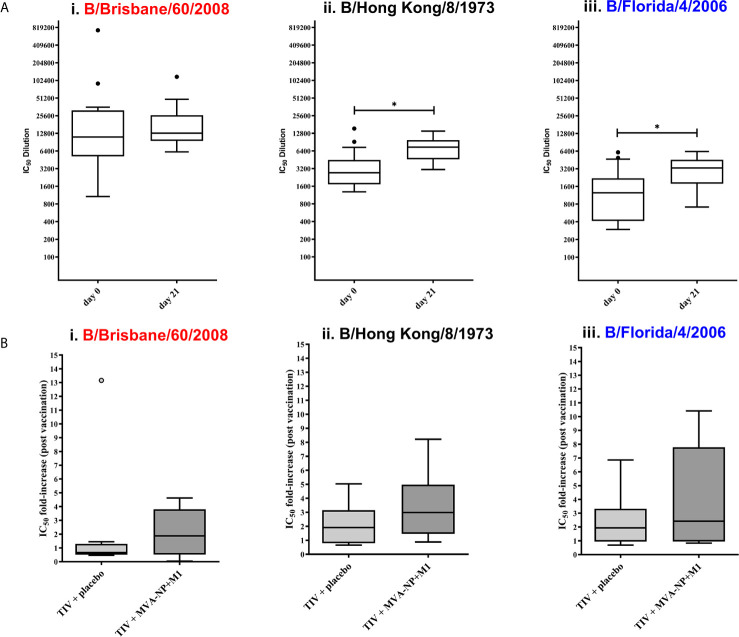
Evaluation of IBV neutralization capacity of NCT0942071 clinical trial sera using influenza B pseudotype microneutralization assay (pMN). The neutralization capacity of human sera from clinical trial (NCT0942071) was assessed pre- and post-vaccination with Trivalent Influenza Vaccine (TIV) and then either a placebo saline solution or an MVA-NP fusion protein (n=17). All sera were serially diluted and incubated with each PV: (i) B/Brisbane/60/2008, (ii) B/Hong Kong/8/1973, and (iii) B/Florida/4/2006 PV before addition of HEK293T/17 cells. Residual luciferase activity was determined and % neutralization was calculated relative to the PV only control. From this, IC50 values were calculated as the reciprocal of the dilution required to neutralize 50% of initial input PV. Significant differences between the means of pre- and post-vaccination sera IC50 when tested against PV are indicated in brackets (*p<0.05) **(A)**. After data stratification on the basis of post-TIV vaccine regimens; TIV + placebo (n=8), TIV+MVA+NP+M1 (n=9), the fold-increase from pre-vaccination IC50 titers were compared to post-vaccination IC_50_ **(B)**. Data is compared using Tukey plots showing the median of the 25^th^ and 75^th^ percentile with outliers plotted as those which exceeded 1.5x quartile range. Virus pseudotype from B/Victoria lineage is indicated in red and B/Yamagata lineage in blue.

Clinical utility: To further investigate the suitability of influenza B pMN assays in vaccine immunogenicity studies and to detect cross−reactive responses to influenza B, we employed human sera from clinical trial NCT00942071 (53). The panel of human sera was provided by Professor Sarah Gilbert (University of Oxford). Using the IBV PV we produced in pI.18, we determined the levels of anti-IBV neutralizing antibodies pre- and post-vaccination with a Trivalent Influenza Vaccine (TIV) (**Figure 5A**). All participants (n=17) had pre-existing antibody response to Flu B – mean IC_50_ dilution of ~61341. These responses were significantly boosted against 2/3 IBV PV tested; B/Hong Kong/8/1973 a pre-split PV (**Figure 5A** (ii) (*p=0.0046* Wilcoxon paired U test) and B/Florida/4/2006, of Yamagata lineage (**Figure 5A** (iii) (*p=0.0129* Wilcoxon paired U test). However, none of the participants had boosted responses against B/Brisbane/60/2008 of Victoria lineage (*p=0.5791* Wilcoxon paired U test). Trial participants received an additional vaccination with either a placebo (saline solution) (n=8) or an MVA-NP-M1 fusion protein (n=9) (53). Antrobus et al. (53) have demonstrated that this additional boost with NP-M1 fusion MVA increased T cell responses to internal influenza proteins. They suggest that this may have helped HA-specific B cells leading to the increased seroprotection and seroconversion to H3N2 they reported (53). Therefore, post vaccination samples were compared between those who had received the NP-M1 fusion MVA and those who received the placebo to determine if this effect was responsible for the increase in post vaccination antibodies observed herein (**Figure 5A**). However, once post-vaccination IC_50_ values were grouped based on the vaccine regimens administered, no significant difference was found in the IC_50_ fold differences between pre-vaccination and post-vaccination values against any of the PV tested (**Figure 5B**). The range of responses was also greater in those participants who received TIV followed by MVA-NP-M1 fusion protein compared to those who received TIV followed by the placebo.

Currently the gold standard for determining vaccine efficacy is to test responses using hemagglutination inhibition (HI) and single radial hemolysis (SRH). We believe that these assays can be complemented by the use of pMN especially in the initial testing of novel vaccine candidate responses. Recently, it has been shown that influenza B pMN fold changes (IC_50_) correlates strongly to SRH and HI assays (62). However, the HI assay is only able to detect antibodies directed against the HA head and we have not differentiated stem responses herein and so this may account for the weak correlation with the HI data presented in the clinical trial (r=0.1632, p=0.3563) (53) (**Supplementary Figure 2**).

Serological surveillance studies monitor the virus and its reservoirs to aid in vaccine choice and treatment. To test whether the pseudotyped IBV could be used to test non-human sera, we obtained seal sera from an influenza surveillance study conducted by the University of Vienna. We evaluated these samples for neutralizing activity against influenza B strains, B/Hong Kong/8/1973, B/Yamagata/16/1988, and B/Victoria/2/1987 from pI.18 (**Figure 2A**) using pMN. In the limited number of sera tested, only a single positive signal was observed, against B/Victoria (IC_50_ = 44) (**Supplementary Table 1**), with none of the other serum samples showing neutralizing activity against any of the IBV PV tested. This correlates with previous data showing a prevalence of antibodies to IBV in only 2% of seals tested (n=971) after 1995 (5).

### Influenza B pMN to Assess Monoclonal Antibody Specificity Against IBV PV From Different Lineages

**Figure 6:**
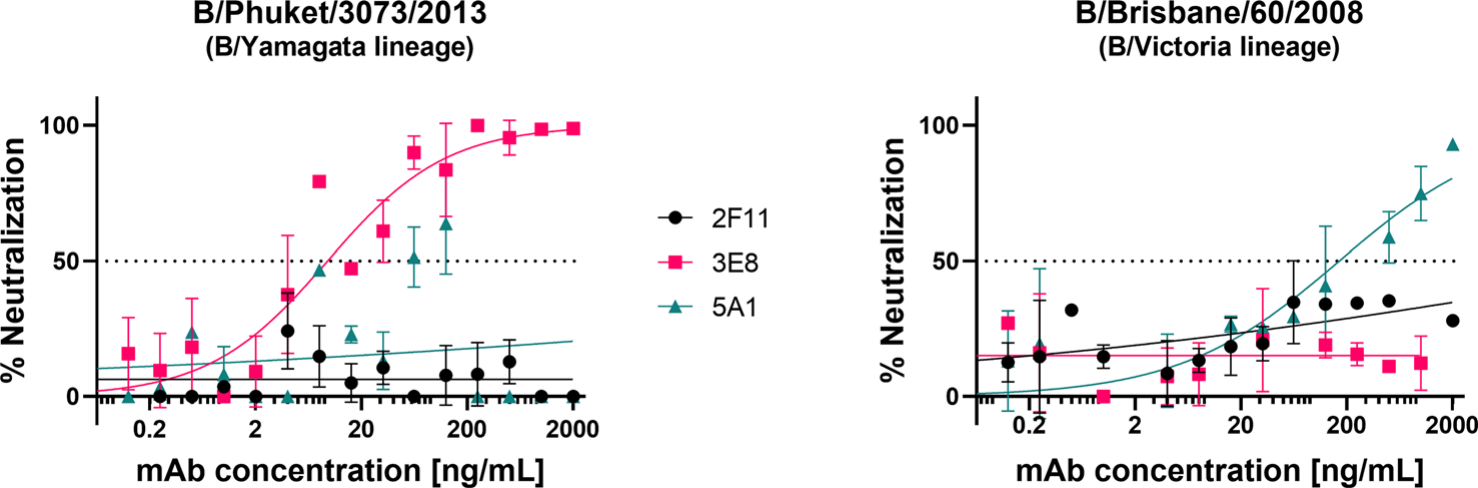
Neutralization of influenza B strains by lineage-specific monoclonal antibodies. Neutralization of influenza B pseudotypes B/Phuket/3073/2013 and B/Brisbane/60/2008 by monoclonal antibodies 3E8, 2F11, and 5A1, was measured by a luciferase reporter assay. Monoclonal antibodies were serially diluted two-fold from a starting concentration ranging from 2000 ng/mL to 0.0625 ng/mL against all pseudotypes tested. Influenza B pseudotypes (1.0x10^6^ RLU) were then added to each well. For all plots, each point represents the mean and standard error of two replicates per dilution.

We tested the influenza B pseudotypes produced from pEVAC-HA (**Figure 2B**) against monoclonal antibodies obtained from the Food and Drug Administration, USA. Monoclonal antibody 3E8, specific to B/Yamagata viruses, was able to neutralize B/Phuket/3073/2013 from the B/Yamagata lineage with an IC_50_ of 18.77 ng/mL (**Figure 6**). However, 3E8 was unable to neutralize the B/Victoria lineage pseudotype. On the other hand, 5A1, specific to B/Victoria viruses, was able to neutralize B/Brisbane/60/2008 from the B/Victoria lineage with an IC_50_ of 206 ng/mL (**Figure 6**). Monoclonal antibody 5A1 was unable to neutralize the B/Yamagata lineage virus. Monoclonal antibody 2F11 which was previously reported to neutralize both B lineages was unable to neutralize both IBV PV at the concentrations tested (**Figure 6**). Our results for 2F11 against B/Brisbane/60/2008 correlate with Carnell, 2021 (62) and we have employed a more recent B/Yamagata-like virus in this study, B/Phuket/3073/2013, compared to those tested previously, which may explain non-neutralization. Epitope mapping of the mAb binding site in these IBV may be done in future to determine if genetic change throughout the years may have influenced neutralization of IBV by this mAb. We have also shown here that neutralization of the IBV pseudotypes we tested was lineage-specific.

## Discussion

Knowledge of the life cycle of the influenza B virus is essential for production of high titer IBV pseudotypes. HA activation through proteolytic cleavage is an integral part of IBV replication. This process of “activation” enables low−pH dependent fusion of the HA envelope with the endosomal membrane leading to endocytosis of the virus, followed by release of the viral core into the cytosol (63). For influenza B, HA activation has been observed in embryonated chicken eggs (64), and in both a trypsin−dependent and independent manner in MDCK cells (65, 66). Additionally, experiments have shown that neuraminidase could also have a role in HA activation by removing glucidic residues on the HA surface (67, 68). Furthermore, it was shown that influenza B HA can be cleaved by porcine pancreatic elastase when the cleavage-linked arginine is substituted with an alanine or a valine (61). Influenza B HA can also partially support a poly−basic cleavage site that permits the cleavage by subtilisin−like proteases (60).

More recently, HAT, TMPRSS2, TMPRSS4, and a range of other transmembrane serine proteases (TSP) have been shown to be able to cleave and activate influenza B HA in *in vitro* models (15, 49, 69). Guided by these findings, we were able to produce high titer IBV PV by including TMPRSS2, TMPRSS4 or HAT protease plasmids in the transfection mix (**Figure 2A**). In the absence of protease (Δprotease), IBV PV are still produced (**Figure 2A**). However, only a small proportion of HA is cleaved by endogenous proteases in the producer HEK293T/17 cells (17). This is illustrated by the presence of the HA1 band, evidence of a cleaved HA0 in Δprotease-produced viruses (**Supplementary Figure 1**). This also further explains the entry of Δ protease PV into HEK293T/17 target cell lines observed during titration experiments. We also utilized exogenous bacterial neuraminidase (NA) for PV production. It has been shown that this NA can play a role in removing glucidic residues from the HA surface, to enhance viral attachment to the receptor (60). However, the addition of exogenous NA to Δ protease PV did not result in heterogeneous glycosylation of HA (**Supplementary Figure 1**). Our data suggest that it is likely that the proteases we employed (especially TMPRSS2) mediate HA cleavage intracellularly (70), *via* interaction with cell glycosylation machinery (71). These findings confirm the importance of host proteases for IBV replication in concurrence with other *in vitro* studies (15, 17, 49, 69).

Here we have shown successful transduction of Influenza B pseudotypes in 3 different cell lines with varying titers (**Figure 3**). It has previously been found that lentiviral particles are produced at high titer in HEK293T cell lines which contain the SV40 T-antigen (72, 73). HEK293T cells have been shown to have shorter doubling times and higher transfection efficiency leading to higher vector titer (74) compared to other cell lines. Several factors influence IBV HA transduction efficacy into target cells. Firstly, different strains of IBV HA bind preferentially to different sialoglycan residues present on the glycoprotein surface of target cells to mediate cell entry (16, 75). Secondly, “hot” cells/cell lines can present different proportions of sialic acid combinations (65, 76). This could be important for transduction into certain cell lines, in relation to differential sialic acid distribution between cells (77). The lipid bilayer and the characteristics of the membrane lipids can also play an important role in transduction as lipid composition has been implicated in membrane fusion (13, 78). Pizzato et al. and Voelkel et al. have observed that lentiviral vector particles are able to bind to the cell membrane and are endocytosed even in absence of envelope proteins (79, 80) and may explain the increased titer in HEK293T/17 cells over MDCK cells (**Figure 3A**). Given these findings, we utilized HEK293T/17 cells as our target cells going forward.

The influenza microneutralization (MN) assay is highly sensitive and specific for detecting virus-specific neutralizing antibodies against influenza viruses (24). However, it is not logistically easy to deal with live influenza viruses that are not widespread in the population, requiring special facilities and training. The use of pseudotyped viruses presents a good alternative to live viruses for research. It was previously reported that neutralizing antibody titers against H5N1 determined by the pseudotype assay (pMN), strongly correlated with titers obtained *via* traditional HI assays using horse erythrocytes and MN assays for human, ferret and avian sera (47). It was also found to be significantly more sensitive for the detection of antibodies to H5 HA than the established method which uses turkey erythrocytes in HI assays (24, 47). In this study, we have utilized the pMN to assess PV sensitivity to reference antisera utilized in vaccine production, vaccine immunogenicity in both mice and human studies, and as a tool in sero-surveillance studies.

The characterization of influenza viruses involves subtype generation and assessment of pathogenicity and is an important step in developing an appropriate regulatory response against the disease. The hemagglutination inhibition (HI) test using reference antisera is the standard method used to subtype influenza (24). Here, we tested the pseudotypes we produced against reference antisera generated by NIBSC that are routinely used to develop vaccine strains. Our results show that the pMN was able to assess the antigenic similarity and/or differences among the influenza B pseudotypes we tested (**Figure 4A**). Antibodies in the anti-B/Brisbane/60/2008 sera were able to neutralize the homologous virus as well as an IBV from the other B lineage and pre-split IBV with differing efficacy (**Figure 4A**). These results are promising, as we can compare antigenic changes in currently circulating influenza viruses with influenza viruses that have circulated in the past to aid in optimal vaccine selection.

Vaccine studies are carried out to demonstrate the ability of various antigens to elicit protective immune responses in different hosts, usually in mice, ferrets, humans, and domestic poultry, as in the case of influenza. Pre-clinical testing of potential vaccine candidates includes *in vitro* assays to check for efficacy and *in vivo* assays using appropriate animal models. We have used our developed pMN assay with IBV PV we produced to test mouse sera vaccinated with various influenza B HA intended to produce an immune response again influenza B. Our study shows that vaccination in mice of B/Colorado/06/2017 and B/Phuket/3073/2013 antigens produced a measurable neutralization response against the IBV pseudotypes tested from both B/Victoria and B/Yamagata lineages that is significantly different from mice vaccinated with PBS (negative control) (**Figure 4B**). IC_50_ dilution values for each antigen against a particular IBV PV can easily be determined from serum titration curves. This makes the pMN using IBV pseudotypes an invaluable tool in testing vaccine immunogenicity and efficacy going forward.

We also evaluated vaccine efficacy using human serum samples from the NCT00942071 clinical trial against B/Hong Kong/8/1973, a PV that has antigenic characteristics typical of influenza B strains circulating pre-lineage split, and B/Florida/4/2006 from the B/Yamagata lineage (**Figure 5A**). The pMN was able to detect neutralizing responses against both PV at all time points tested. Pre-vaccination responses were expected, as the age of the participants in this clinical trial (50 years and above) (53), made these subjects likely to have already encountered the two strains (B/Florida/4/2006 and B/Hong Kong/8/1973) pre-vaccination. Therefore, a certain level of antibodies against these two strains could already be present. However, the high titers observed, especially against the B/Hong Kong/8/1973 PV, and the increase in the neutralization titer after vaccination, indicate a certain level of cross−reactivity.

It was previously shown that seroconversion and concomitant increase in antibody response was observed against a B/Yamagata strain when a trivalent vaccine containing a B/Victoria lineage strain was administered after priming with a Yamagata-strain containing vaccine (81). Similar responses were found herein when neutralization in sera from clinical trial NCT00942071 (53) was assessed *via* the pMN assay. In this trial, a Victoria lineage-based vaccine (B/Brisbane/60/2008) was administered. However, a significant increase in the neutralizing antibody response was only found against a B/Yamagata strain (B/Florida/4/2006) and a pre−lineage split strain (B/Hong Kong/8/1973) and not the B/Victoria lineage vaccine strain. This could indicate that the cross-reactivity between influenza B viruses is primarily related to “antigenic-sin” (82) or that influenza B responses could be driven by antibodies able to cross-react between different influenza B strains.

We further analyzed whether our pMN data had any correlation with the HI data presented in the clinical trial (53). We found weak correlation *(r = 0.1632, p = 0.3563)* between the two data sets (**Supplementary Figure 2**). This is not surprising since the influenza B HI assay has previously been demonstrated to have high variability and low sensitivity (25, 27). It is also known that the HI was unable to detect antigenic differences between influenza B viruses until the 1980’s (8), even with phylogenetic analyses showing that the two distinct lineages were already present in the second half of the 1970’s (7). The differences observed could also be due to the fact that these assays measure different antibody responses (83), with the HI only able to detect responses against the HA head, while the pMN is able to detect both anti-head and anti-stalk neutralizing antibodies (32, 42, 43, 84). It will be necessary to test other sets of sera to better understand correlation between Influenza B pMN and the classic HI assay.

We have also used the pMN assay for influenza surveillance in populations other than humans that may be affected by a zoonotic disease such as influenza. Compared to influenza A, influenza B has only been detected in a limited number of species. Although humans are considered the natural reservoir for IBV, seals have been found to have strains of IBV circulating in approximately 2% of the population (5). The seal sera assessed with the pMN assay here only detected a neutralizing response from one sample which may be due to the low prevalence of IBV in the seal population at the time of sample collection. Therefore, more samples would have to be assessed in strategic locations and timepoints in order to evaluate the potential of the pMN as a screening tool for wildlife surveillance purposes.

Our results demonstrate the ease in which we detected neutralizing activity of lineage-specific monoclonal antibodies against our influenza B pseudotypes. Neutralizing responses of monoclonal antibodies could discriminate between IBV of different lineages. At present, monoclonal antibodies able to cross-neutralize the two influenza B lineages have been isolated from humans and it was shown that they recognize epitopes present at the hinge of the HA head and stalk region or in the stalk region itself (33). However, serological studies have failed to identify these influenza B antibodies mainly as a result of the insensitivity of the classical experimental tools for HA stalk antibody detection. pMN assays can detect responses from both HA-head and HA-stalk directed antibodies. From this standpoint, we have recently shown that the pMN assay strongly correlates with hemagglutination Inhibition and Single Radial Hemolysis when strains from the Yamagata lineage are tested [62]. Further studies should be performed to understand how Influenza B pMN assay correlates with classic microneutralization assays. These studies could also be enhanced by using an influenza B version of an influenza A chimeric PV assay that can delineate functional antibody responses directed against the head from those directed against the stalk (85). The pMN we developed could be further utilized for these purposes.

The data presented in this paper demonstrate the successful production of high titer influenza B pseudotypes using a quadruple plasmid transfection method consisting of HA-containing plasmid, HIV-1 gag-pol packaging construct, luciferase or GFP transfer cassette, and protease, similar to influenza A pseudotype production. The influenza B pseudotypes we have produced here have been shown to measure immune responses against HA with strain and lineage specificity as well as cross-reactivity from various subjects and samples. The influenza B pseudotype neutralization assay can be used to assess potential vaccine candidates, monitor response to vaccination and other interventions, and for surveillance of zoonotic and emerging infections. As such, the next steps will involve further assessing the correlation between pMN and the classic microneutralization and hemagglutination inhibition assays using live virus to establish influenza B sero-protective titers that involve both head and stalk antibodies. These IBV pseudotypes may be indispensable tools that can help minimize the impact of the disease through the use of safe and effective neutralization assays that may guide better influenza control and intervention measures.

## Data Availability Statement

The raw data supporting the conclusions of this article will be made available by the authors, without undue reservation.

## Ethics Statement

The trial protocol was approved within the UK by the Medicines and Healthcare products Regulatory Agency and the Gene Therapy Advisory Committee. The trial was registered at www.clinicaltrials.gov (identifier: NCT00942071).

## Author Contributions

Conceived and designed experiments – FF, JD, KC, GC, SS, and NT. Performed experiments – FF, JD, KC, RK, and GC. Analyzed the data – FF, JD, KC, RK, GC, and NT. Reagent provision – SS, CT, PK, and SG. Wrote the paper – FF, JD, KC, NT. Revised the paper – GC and SS. FF and JD contributed equally to this study. All authors contributed to the article and approved the submitted version.

## Funding

NT, GC, KC, and RK receive funding from the Bill and Melinda Gates Foundation: Grand Challenges Universal Influenza vaccines Award: Ref: G101404. NT and JMD receive funding from Innovate UK, UK Research and Innovation (UKRI) for the project: Digital Immune Optimized and Selected Pan-Influenza Vaccine Antigens (DIOS-PIVa) Award Ref: 105078.

## Conflict of Interest

The authors declare that the research was conducted in the absence of any commercial or financial relationships that could be construed as a potential conflict of interest.

## References

[1] World Health Organization. Influenza Virus Infection in Humans (2014). Available at: https://www.who.int/influenza/human_animal_interface/virology_laboratories_and_vaccines/influenza_virus_infections_humans_feb14.pdf?ua=1.

[2] Petrova VN Russell CA. The Evolution of Seasonal Influenza Viruses. Nat Rev Microbiol (2018) 16(1):47–60. 10.1038/nrmicro.2017.118 29081496

[3] Hause BM Collin EA Liu R Huang B Sheng Z Lu W. Characterization of a Novel Influenza Virus in Cattle and Swine: Proposal for a New Genus in the Orthomyxoviridae Family. mBio (2014) 5(2):1–10. 10.1128/mBio.00031-14 PMC3958797 24595369

[4] Tan J Asthagiri Arunkumar G Krammer F. Universal Influenza Virus Vaccines and Therapeutics: Where Do We Stand With Influenza B Virus? Curr Opin Immunol (2018) 53:45–50. 10.1016/j.coi.2018.04.002 29677684 PMC6141316

[5] Osterhaus AD Rimmelzwaan GF Martina BE Bestebroer TM Fouchier RAM. Influenza B Virus in Seals. Sci (New York NY) (2000) 288(5468):1051 – 3. 10.1126/science.288.5468.1051 10807575

[6] Ran Z Shen H Lang Y Kolb EA Turan N Zhu L. Domestic Pigs are Susceptible to Infection With Influenza B Viruses. J Virol (2015) 89(9):4818. 10.1128/JVI.00059-15 25673727 PMC4403465

[7] Chen R Holmes EC. The Evolutionary Dynamics of Human Influenza B Virus. J Mol Evol (2008) 66(6):655–63. 10.1007/s00239-008-9119-z PMC3326418 18504518

[8] Rota PA Wallis TR Harmon MW Rota JS Kendal AP Nerome K. Cocirculation of Two Distinct Evolutionary Lineages of Influenza Type B Virus Since 1983. Virology (1990) 175(1):59–68. 10.1016/0042-6822(90)90186-U 2309452

[9] Caini S Kusznierz G Garate VV Wangchuk S Thapa B de Paula Júnior FJ. The Epidemiological Signature of Influenza B Virus and Its B/Victoria and B/Yamagata Lineages in the 21st Century. PloS One (2019) 14(9):e0222381–e. 10.1371/journal.pone.0222381 PMC6742362 31513690

[10] Skehel JJ Wiley DC. Receptor Binding and Membrane Fusion in Virus Entry: The Influenza Hemagglutinin. Annu Rev Biochem (2000) 69(1):531–69. 10.1146/annurev.biochem.69.1.531 10966468

[11] Wiley DC Skehel JJ. The Structure and Function of the Hemagglutinin Membrane Glycoprotein of Influenza Virus. Annu Rev Biochem (1987) 56(1):365–94. 10.1146/annurev.bi.56.070187.002053 3304138

[12] Varki A. Diversity in the Sialic Acids. Glycobiology (1992) 2(1):25–40. 10.1093/glycob/2.1.25 1550987 PMC7108601

[13] Chernomordik LV Frolov VA Leikina E Bronk P Zimmerberg J. The Pathway of Membrane Fusion Catalyzed by Influenza Hemagglutinin: Restriction of Lipids, Hemifusion, and Lipidic Fusion Pore Formation. J Cell Biol (1998) 140(6):1369–82. 10.1083/jcb.140.6.1369 PMC2132678 9508770

[14] Garten W Klenk H. Cleavage Activation of the Influenza Virus Hemagglutinin and Its Role in Pathogenesis. .Monogr Virol (2008) 27:156–67. 10.1159/000151618

[15] Laporte M Stevaert A Raeymaekers V Boogaerts T Nehlmeier I Chiu W. Hemagglutinin Cleavability, Acid-Stability and Temperature Dependence Optimize Influenza B Virus for Replication in Human Airways. J Virol (2019) 94(1):JVI.01430–19. 10.1128/JVI.01430-19 PMC6912116 31597759

[16] Wang Y-F Chang C-F Chi C-Y Wang H-C Wang J-R Su I-J. Characterization of Glycan Binding Specificities of Influenza B Viruses With Correlation With Hemagglutinin Genotypes and Clinical Features. J Med Virol (2012) 84(4):679–85. 10.1002/jmv.23219 22337309

[17] Klenk H-D Garten W. Host Cell Proteases Controlling Virus Pathogenicity. Trends Microbiol (1994) 2(2):39–43. 10.1016/0966-842X(94)90123-6 8162439

[18] Glezen WP Schmier JK Kuehn CM Ryan KJ Oxford J. The Burden of Influenza B: A Structured Literature Review. Am J Public Health (2013) 103(3):e43–51. 10.2105/AJPH.2012.301137 PMC3673513 23327249

[19] Dolin R. The Quadrivalent Approach to Influenza Vaccination. J Infect Dis (2013) 208(4):539–40. 10.1093/infdis/jit264 23847061

[20] Beran J Peeters M Dewé W Raupachová J Hobzová L Devaster J-M. Immunogenicity and Safety of Quadrivalent Versus Trivalent Inactivated Influenza Vaccine: A Randomized, Controlled Trial in Adults. BMC Infect Dis (2013) 13(1):224. 10.1186/1471-2334-13-224 23688546 PMC3668902

[21] Pepin S Donazzolo Y Jambrecina A Salamand C Saville M. Safety and Immunogenicity of a Quadrivalent Inactivated Influenza Vaccine in Adults. Vaccine (2013) 31(47):5572–8. 10.1016/j.vaccine.2013.08.069 24016810

[22] Tinoco JC Pavia-Ruz N Cruz-Valdez A Doniz CA Chandrasekaran V Dewé W. Immunogenicity, Reactogenicity, and Safety of Inactivated Quadrivalent Influenza Vaccine Candidate Versus Inactivated Trivalent Influenza Vaccine in Healthy Adults Aged ≥18 Years: A Phase III, Randomized Trial. Vaccine (2014) 32(13):1480–7. 10.1016/j.vaccine.2014.01.022 24486352

[23] Eichner M Schwehm M Hain J Uphoff H Salzberger B Knuf M. 4Flu - an Individual Based Simulation Tool to Study the Effects of Quadrivalent Vaccination on Seasonal Influenza in Germany. BMC Infect Dis (2014) 14(1):365. 10.1186/1471-2334-14-365 24993051 PMC4099094

[24] World Health Organization. Manual for the Laboratory Diagnosis and Virological Surveillance of Influenza (2011). Available at: https://apps.who.int/iris/bitstream/handle/10665/44518/9789241548090_eng.pdf;jsessionid=4568C13F22AAD718DA643A8743196062?sequence=1.

[25] Mancini G Donatelli I Arangio-Ruiz G Rozera C Macchia T. Comparison of Haemagglutination-Inhibition and Single Radial Haemolysis Techniques for Detecting Antibodies to Influenza A and B Viruses. J Hyg (1983) 91(1):157–62. 10.1017/S0022172400060137 PMC2129303 6350442

[26] Trombetta CM Remarque EJ Mortier D Montomoli E. Comparison of Hemagglutination Inhibition, Single Radial Hemolysis, Virus Neutralization Assays, and ELISA to Detect Antibody Levels Against Seasonal Influenza Viruses. Influenza Other Respir Viruses (2018) 12(6):675–86. 10.1111/irv.12591 PMC6185893 30019448

[27] Wood JM Gaines-Das RE Taylor J Chakraverty P. Comparison of Influenza Serological Techniques by International Collaborative Study. Vaccine (1994) 12(2):167–74. 10.1016/0264-410X(94)90056-6 8147099

[28] Kendal AP Cate TR. Increased Sensitivity and Reduced Specificity of Hemagglutination Inhibition Tests With Ether-Treated Influenza B/Singapore/222/79. J Clin Microbiol (1983) 18(4):930. 10.1128/JCM.18.4.930-934.1983 6630472 PMC270933

[29] Monto AS Maassab HF. Ether Treatment of Type B Influenza Virus Antigen for the Hemagglutination Inhibition Test. J Clin Microbiol (1981) 13(1):54. 10.1128/JCM.13.1.54-57.1981 7462419 PMC273720

[30] Pyhälä R Kleemola M Visakorpi R. The HI Test Modified by Ether Treatment in the Sero-Epidemiological Surveillance of Influenza B. J Hyg (1985) 94(03):341–8. 10.1017/S002217240006157X PMC2129485 4008921

[31] Ansaldi F Bacilieri S Amicizia D Valle L Banfi F Durando P. Antigenic Characterisation of Influenza B Virus With a New Microneutralisation Assay: Comparison to Haemagglutination and Sequence Analysis. J Med Virol (2004) 74(1):141–6. 10.1002/jmv.20157 15258980

[32] Corti D Voss J Gamblin SJ Codoni G Macagno A Jarrossay D. A Neutralizing Antibody Selected From Plasma Cells That Binds to Group 1 and Group 2 Influenza A Hemagglutinins. Sci (New York NY) (2011) 333(6044):850–6. 10.1126/science.1205669 21798894

[33] Dreyfus C Laursen NS Kwaks T Zuijdgeest D Khayat R Ekiert DC. Highly Conserved Protective Epitopes on Influenza B Viruses. Science (2012) 337(6100):1343–8. 10.1126/science.1222908 PMC3538841 22878502

[34] Ekiert DC Bhabha G Elsliger MA Friesen RHE Jongeneelen M Throsby M. Antibody Recognition of a Highly Conserved Influenza Virus Epitope. Science (2009) 324(5924):246–51. 10.1126/science.1171491 PMC2758658 19251591

[35] Ekiert DC Friesen RHE Bhabha G Kwaks T Jongeneelen M Yu W. A Highly Conserved Neutralizing Epitope on Group 2 Influenza A Viruses. Science (2011) 333(6044):843–50. 10.1126/science.1204839 PMC3210727 21737702

[36] Sui J Hwang WC Perez S Wei G Aird D Chen L-M. Structural and Functional Bases for Broad-Spectrum Neutralization of Avian and Human Influenza A Viruses. Nat Struct Mol Biol (2009) 16(3):265–73. 10.1038/nsmb.1566 PMC2692245 19234466

[37] Krammer F Palese P Steel J. Advances in Universal Influenza Virus Vaccine Design and Antibody Mediated Therapies Based on Conserved Regions of the Hemagglutinin. In: Oldstone MBA Compans RW, editors. Influenza Pathogenesis and Control vol. II. Cham: Springer International Publishing (2015). p. 301–21. 10.1007/82_2014_408 25007847

[38] Nachbagauer R Feser J Naficy A Bernstein DI Guptill J Walter EB. A Chimeric Hemagglutinin-Based Universal Influenza Virus Vaccine Approach Induces Broad and Long-Lasting Immunity in a Randomized, Placebo-Controlled Phase I Trial. Nat Med (2021) 27(1):106–14. 10.1038/s41591-020-1118-7 33288923

[39] Wiersma LCM Rimmelzwaan GF de Vries RD. Developing Universal Influenza Vaccines: Hitting the Nail, Not Just on the Head. Vaccines (2015) 3(2):239–62. 10.3390/vaccines3020239 PMC4494343 26343187

[40] Yasugi M Kubota-Koketsu R Yamashita A Kawashita N Du A Sasaki T. Human Monoclonal Antibodies Broadly Neutralizing Against Influenza B Virus. PloS Pathog (2013) 9(2):e1003150. 10.1371/journal.ppat.1003150 23408886 PMC3567173

[41] Carnell GW Ferrara F Grehan K Thompson CP Temperton NJ. Pseudotype-Based Neutralization Assays for Influenza: A Systematic Analysis. Front Immunol (2015) 6:161. 10.3389/fimmu.2015.00161 25972865 PMC4413832

[42] Del Rosario JMM Smith M Zaki K Risley P Temperton N Engelhardt OG. Protection From Influenza by Intramuscular Gene Vector Delivery of a Broadly Neutralizing Nanobody Does Not Depend on Antibody Dependent Cellular Cytotoxicity. Front Immunol (2020) 11:627. 10.3389/fimmu.2020.00627 32547534 PMC7273724

[43] Corti D Suguitan Jr AL Pinna D Silacci C Fernandez-Rodriguez MF Vanzetta F. Heterosubtypic Neutralizing Antibodies are Produced by Individuals Immunized With a Seasonal Influenza Vaccine. J Clin Invest (2010) 120: (5):1663–73. 10.1172/JCI41902 PMC2860935 20389023

[44] Liu L Nachbagauer R Zhu L Huang Y Xie X Jin S. Induction of Broadly Cross-Reactive Stalk-Specific Antibody Responses to Influenza Group 1 and Group 2 Hemagglutinins by Natural H7n9 Virus Infection in Humans. J Infect Dis (2017) 215(4):518–28. 10.1093/infdis/jiw608 PMC5853269 28380622

[45] Nachbagauer R Salaun B Stadlbauer D Behzadi MA Friel D Rajabhathor A. Pandemic Influenza Virus Vaccines Boost Hemagglutinin Stalk-Specific Antibody Responses in Primed Adult and Pediatric Cohorts. NPJ Vaccines (2019) 4(1):51. 10.1038/s41541-019-0147-z 31839997 PMC6898674

[46] Cox RJ Mykkeltvedt E Robertson J Haaheim LR. Non-Lethal Viral Challenge of Influenza Haemagglutinin and Nucleoprotein Dna Vaccinated Mice Results in Reduced Viral Replication. Scandinavian J Immunol (2002) 55(1):14–23. 10.1046/j.1365-3083.2002.01015.x 11841688

[47] Temperton NJ Hoschler K Major D Nicolson C Manvell R Hien VM. A Sensitive Retroviral Pseudotype Assay for Influenza H5N1-Neutralizing Antibodies. Influenza Other Respir Viruses (2007) 1(3):105–12. 10.1111/j.1750-2659.2007.00016.x PMC4941878 19453415

[48] Guindon S Gascuel OA. Simple, Fast, and Accurate Algorithm to Estimate Large Phylogenies by Maximum Likelihood. Syst Biol (2003) 52(5):696–704. 10.1080/10635150390235520 14530136

[49] Böttcher E Matrosovich T Beyerle M Klenk HD Garten W Matrosovich M. Proteolytic Activation of Influenza Viruses by Serine Proteases TMPRSS2 and HAT From Human Airway Epithelium. J Virol (2006) 80(19):9896–8. 10.1128/JVI.01118-06 PMC1617224 16973594

[50] Bertram S Glowacka I Blazejewska P Soilleux E Allen P Danisch S. TMPRSS2 and TMPRSS4 Facilitate Trypsin-Independent Spread of Influenza Virus in Caco-2 Cells. J Virol (2010) 84(19):10016–25. 10.1128/JVI.00239-10 PMC2937781 20631123

[51] Naldini L Blömer U Gallay P Ory D Mulligan R Gage FH. In Vivo Gene Delivery and Stable Transduction of Nondividing Cells by a Lentiviral Vector. Science (1996) 272(5259):263. 10.1126/science.272.5259.263 8602510

[52] Ferrara F Temperton N. Pseudotype Neutralization Assays: From Laboratory Bench to Data Analysis. Methods Protoc (2018) 1(1):1–16. 10.3390/mps1010008 PMC6526431 31164554

[53] Antrobus RD Berthoud TK Mullarkey CE Hoschler K Coughlan L Zambon M. Coadministration of Seasonal Influenza Vaccine and MVA-NP+M1 Simultaneously Achieves Potent Humoral and Cell-Mediated Responses. Mol Ther (2013) 22(1):233–8. 10.1038/mt.2013.162 PMC3978791 23831594

[54] Berthoud TK Hamill M Lillie PJ Hwenda L Collins KA Ewer KJ. Potent CD8+ T-Cell Immunogenicity in Humans of a Novel Heterosubtypic Influenza A Vaccine, MVA-NP+M1. Clin Infect Dis (2011) 52(1):1–7. 10.1093/cid/ciq015 21148512 PMC3060888

[55] Verma S Soto J Vasudevan A Schmeisser F Alvarado-Facundo E Wang W. Determination of Influenza B Identity and Potency in Quadrivalent Inactivated Influenza Vaccines Using Lineage-Specific Monoclonal Antibodies. PloS One (2017) 12(4):e0175733–e. 10.1371/journal.pone.0175733 PMC5396888 28423025

[56] Waterhouse AM Procter JB Martin DMA Clamp M Barton GJ. Jalview Version 2–a Multiple Sequence Alignment Editor and Analysis Workbench. Bioinf (Oxford England) (2009) 25(9):1189 – 91. 10.1093/bioinformatics/btp033 PMC2672624 19151095

[57] Edgar RC. MUSCLE: Multiple Sequence Alignment With High Accuracy and High Throughput. Nucleic Acids Res (2004) 32(5):1792–7. 10.1093/nar/gkh340 PMC390337 15034147

[58] Tamura K Peterson D Peterson N Stecher G Nei M Kumar S. MEGA5: Molecular Evolutionary Genetics Analysis Using Maximum Likelihood, Evolutionary Distance, and Maximum Parsimony Methods. Mol Biol Evol (2011) 28(10):2731–9. 10.1093/molbev/msr121 PMC3203626 21546353

[59] Ferrara F Molesti E Böttcher-Friebertshäuser E Cattoli G Corti D Scott SD. The Human Transmembrane Protease Serine 2 Is Necessary for the Production of Group 2 Influenza A Virus Pseudotypes. J Mol Genet Med (2012) 7:309–14. 10.4172/1747-0862.1000055 PMC3614188 23577043

[60] Brassard DL Lamb RA. Expression of Influenza B Virus Hemagglutinin Containing Multibasic Residue Cleavage Sites. Virology (1997) 236(2):234–48. 10.1006/viro.1997.8749 9325231

[61] Stech J Garn H Herwig A Stech O Dauber B Wolff T. Influenza B Virus With Modified Hemagglutinin Cleavage Site as a Novel Attenuated Live Vaccine. J Infect Dis (2011) 204(10):1483–90. 10.1093/infdis/jir613 21917878

[62] Carnell GW Trombetta CM Ferrara F Montomoli E Temperton NJ. Correlation of Influenza B Haemagglutination Inhibiton, Single-Radial Haemolysis and Pseudotype-Based Microneutralisation Assays for Immunogenicity Testing of Seasonal Vaccines. Vaccines (Basel) (2021) 9(2):1–18. 10.3390/vaccines9020100 PMC7911544 33525543

[63] Das DK Govindan R Nikić-Spiegel I Krammer F Lemke EA Munro JB. Direct Visualization of the Conformational Dynamics of Single Influenza Hemagglutinin Trimers. Cell (2018) 174(4):926–37.e12. 10.1016/j.cell.2018.05.050 PMC6086748 29961575

[64] Zhirnov OP Golyando PB Ovcharenko AV. Replication of Influenza B Virus in Chicken Embryos Is Suppressed by Exogenous Aprotinin. Arch Virol (1994) 135(1-2):209–16. 10.1007/BF01309780 7515225

[65] Lugovtsev VY Melnyk D Weir JP. Heterogeneity of the MDCK Cell Line and Its Applicability for Influenza Virus Research. PloS One (2013) 8(9):e75014. 10.1371/journal.pone.0075014 24058646 PMC3772841

[66] Noma K Kiyotani K Kouchi H Fujii Y Egi Y Tanaka K. Endogenous Protease-Dependent Replication of Human Influenza Viruses in Two MDCK Cell Lines. Arch Virol (1998) 143(10):1893–909. 10.1007/s007050050428 9856079

[67] Shibata S Yamamoto-Goshima F Maeno K Hanaichi T Fujita Y Nakajima K. Characterization of a Temperature-Sensitive Influenza B Virus Mutant Defective in Neuraminidase. J Virol (1993) 67(6):3264–73. 10.1128/JVI.67.6.3264-3273.1993 PMC237667 8497050

[68] Yamamoto-Goshima F Maeno K. Approach to the Involvement of Influenza B Neuraminidase in the Cleavage of HA by Host Cell Protease Using Low Ph-Induced Cell Fusion Reaction. Microbiol Immunol (1994) 38(10):819–22. 10.1111/j.1348-0421.1994.tb01864.x 7869962

[69] Limburg H Harbig A Bestle D Stein DA Moulton HM Jaeger J. Tmprss2 Is the Major Activating Protease of Influenza A Virus in Primary Human Airway Cells and Influenza B Virus in Human Type II Pneumocytes. J Virol (2019) 93(21):e00649–19. 10.1128/JVI.00649-19 PMC6803253 31391268

[70] Böttcher E Freuer C Steinmetzer T Klenk H-D Garten W. MDCK Cells That Express Proteases TMPRSS2 and HAT Provide a Cell System to Propagate Influenza Viruses in the Absence of Trypsin and to Study Cleavage of HA and its Inhibition. Vaccine (2009) 27(45):6324–9. 10.1016/j.vaccine.2009.03.029 19840668

[71] Walker JA Sakaguchi T Matsuda Y Yoshida T Kawaoka Y. Location and Character of the Cellular Enzyme That Cleaves the Hemagglutinin of a Virulent Avian Influenza Virus. Virology (1992) 190(1):278–87. 10.1016/0042-6822(92)91214-f 1529533

[72] Lu X Humeau L Slepushkin V Binder G Yu Q Slepushkina T. Safe Two-Plasmid Production for the First Clinical Lentivirus Vector That Achieves >99% Transduction in Primary Cells Using a One-Step Protocol. J Gene Med (2004) 6(9):963–73. 10.1002/jgm.593 15352069

[73] Naldini L Blömer U Gage FH Trono D Verma IM. Efficient Transfer, Integration, and Sustained Long-Term Expression of the Transgene in Adult Rat Brains Injected With a Lentiviral Vector. Proc Natl Acad Sci (1996) 93(21):11382. 10.1073/pnas.93.21.11382 8876144 PMC38066

[74] Merten O-W Charrier S Laroudie N Fauchille S Dugué C Jenny C. Large-Scale Manufacture and Characterization of a Lentiviral Vector Produced for Clinical Ex Vivo Gene Therapy Application. Hum Gene Ther (2010) 22(3):343–56. 10.1089/hum.2010.060 21043787

[75] Matrosovich MN Gambaryan AS Tuzikov AB Byramova NE Mochalova LV Golbraikh AA. Probing of the Receptor-Binding Sites of the H1 and H3 Influenza A and Influenza B Virus Hemagglutinins by Synthetic and Natural Sialosides. Virology (1993) 196(1):111–21. 10.1006/viro.1993.1459 8356788

[76] Varki NM Varki A. Diversity in Cell Surface Sialic Acid Presentations: Implications for Biology and Disease. Lab Invest (2007) 87(9):851–7. 10.1038/labinvest.3700656 PMC7100186 17632542

[77] Sieben C Kappel C Zhu R Wozniak A Rankl C Hinterdorfer P. Influenza Virus Binds its Host Cell Using Multiple Dynamic Interactions. Proc Natl Acad Sci (2012) 109(34):13626–31. 10.1073/pnas.1120265109 PMC3427095 22869709

[78] Heaton NS Randall G. Multifaceted Roles for Lipids in Viral Infection. Trends Microbiol (2011) 19(7):368–75. 10.1016/j.tim.2011.03.007 PMC3130080 21530270

[79] Pizzato M Marlow SA Blair ED Takeuchi Y. Initial Binding of Murine Leukemia Virus Particles to Cells Does Not Require Specific Env-Receptor Interaction. J Virol (1999) 73(10):8599. 10.1128/JVI.73.10.8599-8611.1999 10482613 PMC112880

[80] Voelkel C Galla M Dannhauser PN Maetzig T Sodeik B Schambach A. Pseudotype-Independent Nonspecific Uptake of Gammaretroviral and Lentiviral Particles in Human Cells. Hum Gene Ther (2012) 23(3):274–86. 10.1089/hum.2011.011 22010882

[81] Skowronski DM Hamelin M-E Janjua NZ Serres GD Gardy JL Rhéaume C. Cross-Lineage Influenza B and Heterologous Influenza A Antibody Responses in Vaccinated Mice: Immunologic Interactions and B/Yamagata Dominance. PloS One (2012) 7(6):e38929. 10.1371/journal.pone.0038929 22745690 PMC3382187

[82] Francis T. (1960). On the Doctrine of Original Antigenic Sin. JSTOR, in: Proceedings of the American Philosophical Society. p. 572–8.

[83] Molesti E Ferrara F Lapini G Montomoli E Temperton NJ. Discordant Correlation Between Serological Assays Observed When Measuring Heterosubtypic Responses Against Avian Influenza H5 and H7 Viruses in Unexposed Individuals. BioMed Res Int (2014) 2014(5):231365–12. 10.1155/2014/231365 PMC4071775 25013769

[84] Ramage W Gaiotto T Ball C Risley P Carnell GW Temperton N. Cross-Reactive and Lineage-Specific Single Domain Antibodies Against Influenza B Hemagglutinin. Antibodies (Basel) (2019) 8(1):1–20. 10.3390/antib8010014 PMC6640691 31544820

[85] Ferrara F Temperton N. Chimeric Influenza Haemagglutinins: Generation and Use in Pseudotype Neutralization Assays. MethodsX (2017) 4:11–24. 10.1016/j.mex.2016.12.001 28070500 PMC5219643

