# Supplementary material for: Development of Lentiviral Vectors Pseudotyped With Influenza B Hemagglutinins: Application in Vaccine Immunogenicity, mAb Potency, and Sero-Surveillance Studies
Source: Front Immunol. 2021 May 24;12:661379. doi: 10.3389/fimmu.2021.661379 (PMC8182064; doi:10.3389/fimmu.2021.661379)
Supplement: Supplementary file 1 [file DataSheet_1.docx]

Supplementary Material


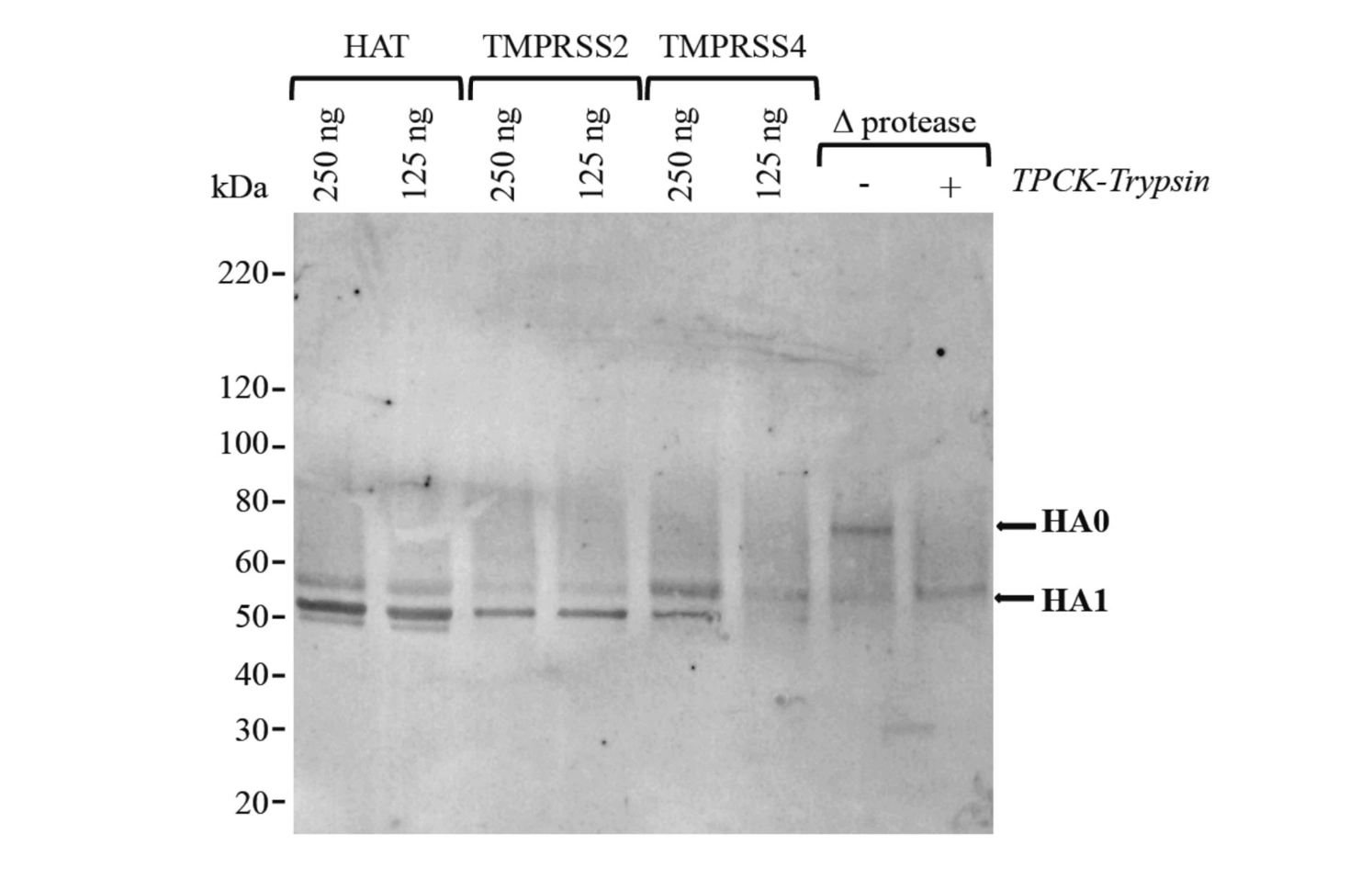


**Supplementary Figure 1.** **Cleavage of influenza B pseudotype Hemagglutinin.** Detection of HA0 and hemagglutinin cleavage product HA1 after PV production employing proteases HAT, TMPRSS2, and TMPRSS4 was done via western blotting. No protease (Δprotease) and TPCK-trypsin treated IBV pseudotypes served as controls. Theoretical protein sizes for HA0 are ~78 kDA and HA1 ~50-58 kDA.


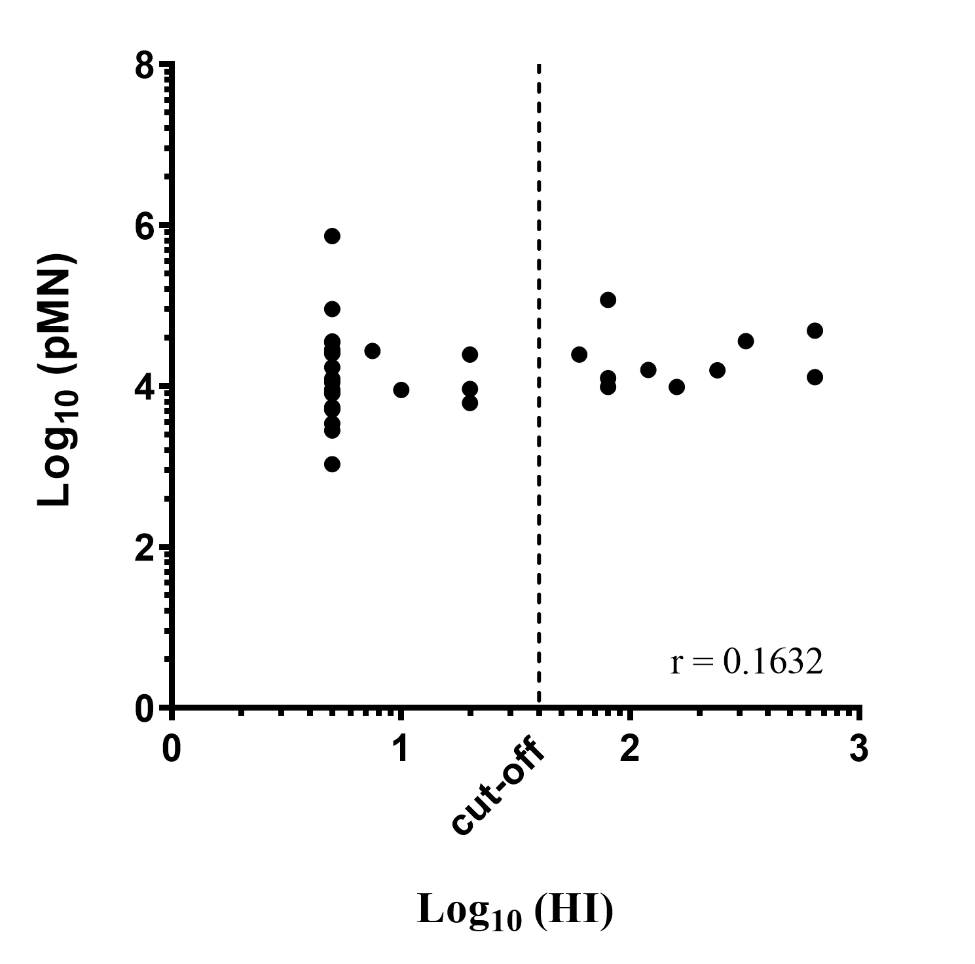


**Supplementary Figure 2.** Correlation of HI and pMN assays performed on serum samples from the NCT00942071 clinical trial (Antrobus, Berthoud et al. 2013). Pseudotype microneutralization assays were performed with the B/Brisbane/60/2008 influenza PV produced in the study. HI titers were taken from clinical trial data. The cut off reported on the log10 (HI) axis corresponds to log10 (40).

**Supplementary Table 1.** Use of influenza B PV in neutralization assays utilizing seal sera. Values are reported as reciprocal dilution values for serum to neutralize 50% of input PV (IC_50_). Grey cells indicate no neutralization.

| **Serum Sample** | | **Influenza B Pseudotyped Virus (PV)** | | |
| --- | --- | --- | --- | --- |
|  |  | B/HongKong/8/1973 | B/Yamagata/16/1988 | B/Victoria/2/1987 |
|  | **9** |  |  |  |
|  | **15** |  |  |  |
|  | **16** |  |  |  |
| Seal from Caspian Sea | **17** |  |  |  |
|  | **20** | 11 |  |  |
|  | **22** |  |  |  |
|  | **23** |  |  | 44 |
|  | **25** |  | 2 |  |
| Positive control  anti-flu B  (NIBSC, Sheep) |  | 7921 | 6541 | 7729 |
| Positive control (human) | **304(21)** | 150 | 426 | 44 |
| Negative control | **FBS** |  |  |  |
